# Genotype and Age at Onset Drive Vermis Atrophy in *CACNA1A*- and GAA-*FGF14*-related Ataxias

**DOI:** 10.1007/s12311-026-01966-8

**Published:** 2026-03-04

**Authors:** Elisabetta Indelicato, Wolfgang Nachbauer, Matthias Amprosi, David Pellerin, Stephanie Mangesius, Elke R. Gizewski, Stefan Kiechl, Bernhard Brais, Sylvia Boesch, Florian Krismer

**Affiliations:** 1 Center for Rare Movement Disorders Innsbruck, Department of Neurology, Medical University Innsbruck, Anichstrasse 35, 6020 Innsbruck, Austria; 2 Department of Neurology and Neurosurgery, Montreal Neurological Institute, McGill University, Montreal, QC Canada; 3 Dr. John T. Macdonald Foundation Department of Human Genetics and John P. Hussman Institute for Human Genomics, University of Miami Miller School of Medicine, Miami, FL 33101 USA; 4 Department of Radiology, Medical University of Innsbruck, 6020 Innsbruck, Austria; 5 Neuroimaging Research Core Facility, Medical University of Innsbruck, Anichstraße 35, 6020 Innsbruck, Austria; 6 VASCage, Centre On Clinical Stroke Research, Innsbruck, Austria

**Keywords:** *CACNA1A*, Cerebellum, CerebNet, Computational Neuroimaging, GAA-*FGF14* related ataxia, Spinocerebellar Ataxia 27B

## Abstract

*CACNA1A*- and GAA-*FGF14*-related channelopathies are among the most frequent genetic etiologies of cerebellar ataxia. They display overlapping features consisting of both chronic and episodic neurological symptoms and usually show only a mild cerebellar atrophy in imaging studies, with prevalent involvement of the vermis. To evaluate cerebellar volumetry as a biomarker for distinguishing between these disorders and for understanding their neuroanatomical correlates. We applied a deep learning method (CerebNet) for the lobular segmentation and volumetry assessment of the cerebellum on 3.0 Tesla MRI scans of patients with genetically confirmed *CACNA1A*- (n = 16) and GAA-*FGF14*-related (n = 12) ataxia. K-means clustering and principal component analysis were employed to assess infratentorial atrophy patterns. Three distinct clusters based on the patterns of infratentorial volume loss were established, with vermian atrophy contributing the most. The degree of vermian atrophy was not correlated with the clinical severity of chronic ataxia, but with age at disease onset (*r*_*s*_(26) = 0.47, *p* = 0.01). The cluster with most marked atrophy of the vermis comprised patients with missense *CACNA1A* variants who exhibited an early disease onset and migraine with aura as episodic manifestation. Conversely, there was an increase in the frequency of episodic ataxia and both loss-of-function *CACNA1A* variants and GAA-*FGF14*-expansions as genotypes when progressing from clusters with more to less severe vermian atrophy. Age at onset and the modality of channel dysfunction are key determinants of cerebellar volume loss in *CACNA1A* and *GAA-FGF14*-disease.

**Supplementary Information:**

The online version contains supplementary material available at 10.1007/s12311-026-01966-8.

## Introduction

Ion channel dysfunction is a recurring etiology of inherited ataxias [1]. Ataxic syndromes associated to channelopathies typically present with both episodic and chronic neurological symptoms and usually show a prevalent or exclusive involvement of the vermis in imaging studies [1–4]. These features are recapitulated by *CACNA1A*- [OMIM *601011] and *FGF14*- [OMIM *601515] related disorders, which list among the most frequent molecular etiologies of inherited ataxias [5, 6].

*CACNA1A* is a bicistronic gene which encodes both the pore-forming α1A-subunit of the neuronal P/Q Ca^2+^ channel and the transcription factor α1ACT which drives maturation of the Purkinje cells in the early development [7]. Single nucleotide variants and small deletions in *CACNA1A* are associated with dominantly inherited phenotypes sharing a common denominator of chronic cerebellar signs and paroxysmal features [2]. Classical episodic manifestations associated with *CACNA1A* disease spectrum include hemiplegic migraine, which is typically associated with missense variants (OMIM #141500, “Familial Hemiplegic Migraine 1”), and episodic ataxia, which are more often observed in cases with loss-of-function variants (OMIM #108500, “Episodic Ataxia Type 2”) [8]. Clinical features associated with *CACNA1A* variants can vary greatly, even within the same family. Age at onset can range from the first to the seventh decade [5].

*FGF14* encodes a member of the fibroblast growth factor family, which has been shown to directly bind and regulate potassium and voltage-gated sodium channels, thus influencing membrane potential and synaptic transmission [9, 10]. Recently, a pathologically expanded deep intronic GAA•TTC repeat in the *FGF14* gene has been associated with a dominantly inherited adult-onset ataxia with frequent episodic features, also known as spinocerebellar ataxia 27B (SCA27B, OMIM #620,174) [6]. Cumulative evidence suggests that this entity may become one of the most frequent inherited ataxias as the genetic testing becomes widely available [11].

From a clinical perspective, phenotypes of adult patients with GAA-*FGF14*- and *CACNA1A*-related episodic ataxia largely overlap [12]. Sparse literature describes either unremarkable brain MRI or a mild cerebellar atrophy, usually restricted to the vermis in both disorders [13–15].

With these entities being increasingly recognized, a growing interest is focusing on the definition of biomarkers which may support differential diagnosis, reflect prognostic trajectories and clinical course, with potential application in future trials. Cerebellar volume estimation from structural MRI is a relevant neuroanatomical marker which has been intensively studied in other inherited cerebellar ataxias [16–20]. In *CACNA1A* disorders, systematic imaging studies are limited to single cohorts studied with MR spectroscopy [21, 22]. One study identified hyperintensities of the superior cerebellar peduncles on MRI as a characteristic, albeit modestly sensitive, sign of GAA-*FGF14*-related ataxia [15].

In the present study, we applied a deep learning method for the lobular segmentation of the cerebellum [16] to assess cerebellar volumetry in a cohort of patients with cerebellar ataxia due to either non-polyglutamine *CACNA1A* variants or GAA-*FGF14*-expansions. Our objectives were i) to evaluate the diagnostic performance and discriminative power of this novel methodology and ii) to identify molecular and clinical correlates associated with the neuroanatomical mapping-based findings.

## Patients and Methods

### Study Population

Adult patients with cerebellar ataxia and genetically confirmed non-polyglutamine *CACNA1A* disease (from now on referred to as “*CACNA1A* disease”) or GAA-*FGF14*-related disease were recruited at the Center for Rare Movement Disorders of the Medical University of Innsbruck. The diagnosis of *CACNA1A* disease was confirmed based on the detection of a variant that was classified as likely pathogenic or pathogenic according to the criteria of the American College of Medical Genetics and Genomics (ACMG) [23]. The diagnosis of SCA27B was confirmed based on the detection of a deep intronic GAA•TTC expansion in *FGF14* of at least 250 uninterrupted repeats. GAA-*FGF14*-expansions testing was performed according to a previously described protocol [24]. The severity of the cerebellar syndrome was defined by means of the SARA (Scale for the Assessment and Rating of Ataxia) score [25].

### Imaging Studies and Statistical Analysis

MRI acquisitions were performed on a 3.0 Tesla whole‐body Siemens MR scanner. 3-dimensional T1‐weighted images were processed with the FastSurfer pipeline [26] and the CerebNet module [16] to segment subcortical brain region and estimate different brain regions volumes. Normative data for subcortical regional volumes over the lifetime of the adult human brain were generated from 236 healthy participants from open access MR datasets from the Parkinson’s Progression Markers Initiative (PPMI; for up to date information see https://www.ppmi-info.org/) and National Institute of Mental Health (NIMH) Data Archive (https://nda.nih.gov/, dataset 10.18112/openneuro.ds005752.v2.1.0) in compliance with the respective data use agreements. Data used in the preparation of this article were also obtained from the Alzheimer’s Disease Neuroimaging Initiative (ADNI) database (adni.loni.usc.edu). The ADNI was launched in 2003 as a public–private partnership, led by Principal Investigator Michael W. Weiner, MD. The primary goal of ADNI has been to test whether serial magnetic resonance imaging (MRI), positron emission tomography (PET), other biological markers, and clinical and neuropsychological assessment can be combined to measure the progression of mild cognitive impairment (MCI) and early Alzheimer’s disease (AD).

We performed statistical analyses with the IBM software SPSS version 29 and R version 4.5.0. Generalized Additive Models were employed using the GAMLSS package in R to investigate the influence of age and sex on regional brain volumes in healthy adults. A separate model was fitted for each brain region of interest, with the respective volume measure as the dependent variable. The models included age (modelled non-linearly using penalized B-splines with three degrees of freedom) and sex as predictors. For patients, individual deviations from the normative distribution were reported as z-scores. To this end, the parameters of the GAMLSS models previously fitted based on healthy controls were used to estimate the expected distribution (μ, σ, ν, and τ) for a given age and sex. The cumulative distribution function of the Box–Cox power exponential (BCPE) distribution was evaluated at the patient's observed volume and the resulting percentile was converted into a standard z-score. After z-transformation, a k-means clustering analysis with 3 centers was performed to group the data points into distinct clusters based on their similarity, aiming to identify underlying patterns of infratentorial brain atrophy. Subsequently, a principal components analysis (PCA) of the cluster dimensions was conducted to study what dimensions primarily drive cluster separation. Comparisons among clusters were conducted using one-way ANOVA or the Kruskal–Wallis test, with Bonferroni correction applied for multiple comparisons as appropriate. Spearman’s rank correlation coefficient was used to assess correlations.

## Results

### Clinical Characteristics

The patient cohort addressed in this study consisted of 28 adults with cerebellar ataxia, including 16 diagnosed with *CACNA1A*-related disease and 12 with GAA-*FGF14*-disease. Demographic and clinical characteristics are reported in Table 1. Patients with *CACNA1A* disease were younger and had an earlier age at onset of disease. The severity of chronic ataxia was mostly mild and comparable in patients with *CACNA1A* disease and GAA-*FGF14*-ataxia. Clinical history revealed spells of episodic ataxia in 7/16 (44%) patients with *CACNA1A* disease and 11/12 (92%) patients with GAA-*FGF14*-disease. (*p* = 0.01 in the comparison). Migraine with aura was reported exclusively by patients with *CACNA1A* disease (in 11/16, 44% of cases, *p* < 0.001 in the comparison with GAA-*FGF14*-disease). At the time of the MRI, 14/16 patients with *CACNA1A* disease and 3/12 patients with GAA-*FGF14*-disease assumed an interval prophylaxis. Involvement of the superior cerebellar peduncle, as observed on T2-weighted MRI sequences [27] was detected in 4 of 12 patients with GAA-*FGF14*-disease (33%), appearing prominently in two patients and faintly in the remaining two. This sign was not observed in any patients with *CACNA1A* disease (*p* = 0.024 in the comparison).

**Table 1:** Demographics and clinical characteristics of the study cohort

	*CACNA1A* (n = 16)	GAA-*FGF14* (n = 12)	*p value*
Gender n. of females, %	4 (25%)	5 (42%)	0.299*
Age at MRI years, median (IQR)	56 (38–64)	70 (63–73)	0.002**
SARA score at MRI median (IQR)	9 (6–12)	11 (8–17)	0.698**
SCP sign n., %	0	4 (33%)	0.024*
Phenotype			
Migraine with aura n., %	11 (69%)	0 (0%)	< 0.001*
Episodic Ataxia n., %	7 (44%)	11 (92%)	0.011*
Age at onset of episodic symptoms years, median (IQR)	12 (5–24)	58 (50–70)	< 0.001**
Age at onset of chronic ataxia years, median (IQR)	38 (19–54)	61 (54–70)	< 0.001**
Interval therapy			
Acetazolamide n., %	7 (44%)	3 (25%)	
4-aminopyridine n., %	3 (19%)	1 (8%)	
Topiramate n., %	2 (13%)	0	
Flunarizine n., %	2 (13%)	0	
No interval therapy n., %	2 (13%)	10 (83%)	

*IQR* interquartile range; *SARA* Scale for the Assessment and Rating of Ataxia; *SCP* superior cerebellar peduncle. *P* values were calculated using the *Fisher's exact test or the **Mann–Whitney U test

### Cluster analysis of Cerebellar Lobules Volumetry

**Fig. 1:**
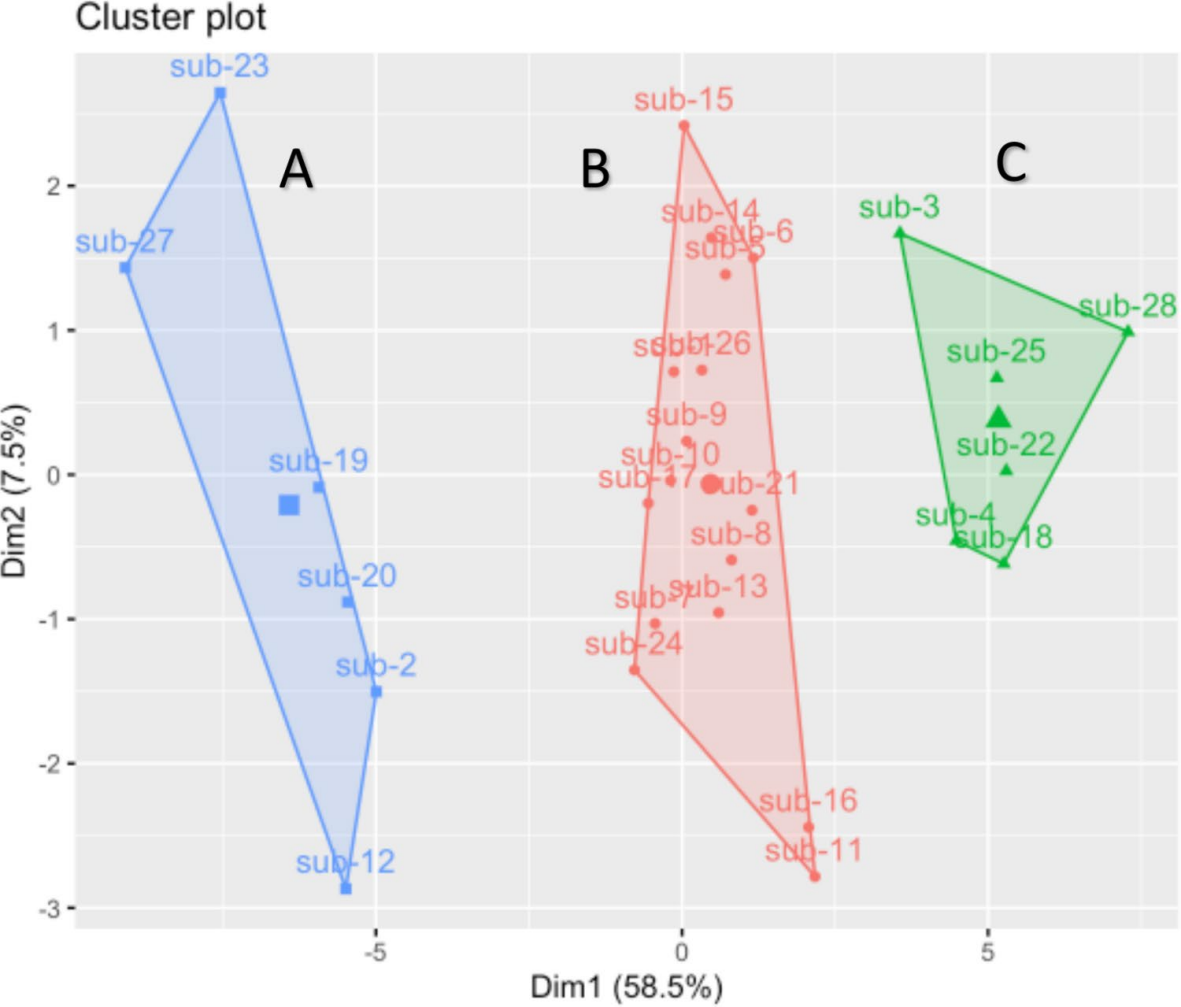
Clustering by principal component analysis of cerebellar lobules volumetry

The k-means clustering analysis defined three clusters based on the pattern of infratentorial brain atrophy, as shown in Fig. 1. The PCA revealed that the first principal component explained 58.5% of the variance indicating that it captured the most significant patterns. Vermis atrophy as well as the cerebellar lobules IV – VII had the highest relative contribution to this principal component. Cluster A (blue labelled in Fig. 1) comprised subjects with the highest degree of atrophy of the cerebellar vermis (z score > 4 in the comparison with controls). Cluster B and C (red and green labelled respectively in Fig. 1) could not be distinguished from controls based solely on the degree of vermian atrophy. Cluster A comprised six subjects, who all carried missense variants in *CACNA1A*. Cluster B comprised 16 patients of whom eight with GAA-*FGF14*-expansions and eight with *CACNA1A* variants (six missense, two loss-of-function). Cluster C comprised six patients, also with a mixed genotype (four GAA-*FGF14*-expansions and two *CACNA1A* variant of which one missense and one loss-of-function). Detailed patients’ clinical data are available in the supplementary material. Regarding the phenotype, all patients from cluster 1 presented migraine with aura as main paroxysmal manifestation (*p* = 0.004 in the groups comparison, see also Table 2). Conversely, the frequency of episodic ataxia showed an opposite trend with increasing frequency from cluster A to cluster C (*p* = 0.028). Comparison across the three groups did not show statistically significant differences concerning the age at the time of MRI. Instead, cluster A patients displayed a significantly lower age at onset of both episodic symptom and chronic ataxia in comparison to the other two clusters (*p* = 0.010 and *p* = 0.023, respectively). In the whole cohort, the age at onset of chronic ataxia displayed a significant, moderate correlation with the degree of vermian atrophy (as expressed by the z scores, *r*_*s*_(26) = 0.47, *p* = 0.01). The severity of chronic cerebellar syndrome (as expressed by the SARA score) at the time of the MRI was comparable across the three groups and did not display a correlation with the degree of vermian atrophy.

**Table 2:** Volumetry-based group stratification: demographics and clinical characteristics

	*Cluster A (n* = *6)*	*Cluster B* (n = 16)	*Cluster C* (n = 6)	*p value*
Age at MRI years, 95%(CI)	49 (36–62)	63 (55–70)	58 (39–76)	0.119*
SARA score at MRI median (IQR)	10 (7–12)	11 (9–15)	7 (4–10)	0.125*
*CACNA1A* genotype n.,%	6 (100%)	8 (50%)	2 (33%)	0.035**
Phenotype				
Migraine with Aura n., %	6 (100%)	4 (25%)	1 (17%)	0.004**
Episodic Ataxia n., %	1 (17%)	12 (75%)	5 (83%)	0.028**
Age at onset of episodic symptoms years, median (IQR)	6 (2–12)	55 (14–63)	49 (11–66)	0.010*
Age at onset of chronic ataxia years, median (IQR)	23 (15–44)	58 (46–65)	56 (42–70)	0.023*

*IQR* interquartile range; *SARA* Scale for the Assessment and Rating of Ataxia. *P* values were calculated using the *one-way ANOVA or the ** Kruskal–Wallis test, with Bonferroni correction applied for multiple comparisons

Based on cerebellar lobules volumetry patients were stratified in three clusters with increasing infratentorial atrophy (A > B > C). Detailed clinical data on each subject are provided in the supplement.

## Discussion

In many ataxic disorders, cerebellar atrophy represents a relevant non-invasive neuroanatomical marker [17–19] whose evolution can be accurately captured via volume estimation from structural MRI, already in a pre-symptomatic disease stage [19, 28]. Cerebellar atrophy is a proxy of disease severity in this setting [17, 18, 29, 30].

*CACNA1A*- and GAA-*FGF14*-related disorders are among the most frequent genetic causes of cerebellar ataxia. *CACNA1A*- and GAA-*FGF14*-related disorders are the main differential diagnoses to consider when chronic cerebellar signs go along with episodic neurological manifestations. In contrast to other cerebellar disorders, they are usually accompanied by a rather mild cerebellar atrophy at MRI which, if present, is mostly limited to the vermis [13, 14]. In the clinical experience, visual inspection of brain MRI has limited sensitivity in detecting atrophy changes related to *CACNA1A* disease and GAA-*FGF14*-related ataxia as well as in quantifying their evolution over time. As a result, the application of cerebellar volumetry is of utmost interest and an unmet need in the quest for biomarkers in these disorders [15, 31]. Bearing this in mind, we applied a recently described deep learning method [16] to assess cerebellar volumetry in a cohort of patients with *CACNA1A* disease and GAA-*FGF14*-related ataxia. We made a few key observations. First, automated cerebellar volumetry clearly separated patients based on the degree of vermian atrophy. Secondly, this neuroanatomical marker neither did correlate with the age at the examination nor, more interestingly, with the severity of the chronic cerebellar syndrome. Instead, migraine with aura and the age at disease onset were the clinical features which showed the strongest association with the volumetric findings. Notably, the cluster exhibiting the most pronounced vermian atrophy consisted of patients with homogeneous characteristics, including early-onset ataxia, migraine with aura, and the presence of missense *CACNA1A* variants. Progressing from clusters with more to less severe vermian atrophy, there was a corresponding increase in the frequency of episodic ataxia as phenotype and both loss-of-function *CACNA1A* variants and GAA-*FGF14*-expansions as genotypes. Taken together these observations reflect a correlation of atrophy-based clustering with the underlying form of channel dysfunction. In fact, a gain-of-function mechanism underlies the phenotype of missense *CACNA1A* variants resulting in migraine with aura [32], whereas a loss-of-function mechanism is predicted to underlie episodic ataxia, both in the context of frameshift/truncating *CACNA1A* variants and in GAA-*FGF14* expansions [6]. In murine models, both loss-of-function variants in *CACNA1A* and *FGF14* disrupt the pacemaking function of the Purkinje cell and trigger paroxysmal motor symptoms [33, 34].

A tight correlation between the age at onset and the clinical course of *CACNA1A* disorders has been already observed and a phenotypic “spectrum” can be delineated based on the age at onset [35]. At the one extreme of this spectrum are early onset, most severe phenotypes such as “developmental encephalopathies”, which are associated with missense gain-of-function variants arising de novo [36]. The present MRI findings reinforce the concept of an age dependency of the *CACNA1A* phenotype [35], whose underlying pathophysiological mechanisms await to be unveiled. At central synapses, including the inhibitory synapses between Purkinje cells and deep cerebellar nuclei neurons, there is a developmental shift in the Ca^2^⁺ currents that mediate synaptic transmission [37]. This shift results in an increased reliance on P/Q-type calcium channels as postnatal development progresses [37]. Additionally, the expression of specific splicing variants of P/Q-type channels varies with age [38]. In this context, specific disease-causing variants in *CACNA1A* may have a particularly detrimental impact on the developing brain, leading to an enhanced susceptibility to neurodegeneration, as detected by automated cerebellar volumetry. Longitudinal studies would be crucial in determining whether these findings evolve across the lifespan and whether they correlate with the progression of clinical symptoms.

The discrepancy between the clinical severity of ataxia in some patients and the mild neuroanatomical alterations at MRI suggest that neurodegeneration is not a main or the sole- driver of cerebellar dysfunction in these disorders. This gives rise to hope for the definition of therapeutic strategies which might revert part of the neurological symptoms. To this concern, cerebellar volumetry may be helpful in stratifying patients for clinical studies by identifying those with a less prominent neurodegeneration who thus may benefit from early neuroprotective trials.

The present study bears several limitations. Cerebellar volumetry is currently used only in research settings and has not yet been integrated as a clinical tool in the diagnostic work-up of ataxias. The small sample size limits the generalizability of our conclusions. To address this limitation, future studies with larger cohorts are needed to corroborate our findings and to further investigate potential correlations between clinical milestones and imaging markers. If applied to a larger cohort, automated cerebellar volumetry could further validate the selective pattern of atrophy highlighted in this study and support its use as an additional diagnostic clue in the clinical work-up.

## Conclusion

In conclusion, the pattern of cerebellar vermis atrophy as determined via automated cerebellar volumetry in *CACNA1A-* and GAA-*FGF14*-related disorders do not correlate with the severity of ataxia. Instead, the age at onset and the molecular mechanism of channel dysfunction were the most important determinants of vermian volume loss in our study. Replication and complementation with further experimental studies are advocated to unveil the pathophysiology of cerebellar dysfunction in these disorders.

In the future, early application of automated cerebellar volumetry to routine MRI scans may help detect visually unremarkable, region-specific patterns of cerebellar atrophy that are consistent with *CACNA1A*- and GAA-*FGF14*-related disorders.

## Supplementary Information

Below is the link to the electronic supplementary material.

Supplementary file1 (DOCX 19 KB)

## Data Availability

The dataset supporting the present findings is available from the corresponding author upon reasonable request.

## References

[1] Coutelier M, Coarelli G, Monin ML, Konop J, Davoine CS, Tesson C, et al. A panel study on patients with dominant cerebellar ataxia highlights the frequency of channelopathies. Brain. 2017;140(6):1579–94. 28444220 10.1093/brain/awx081

[2] Indelicato E, Boesch S. CACNA1A-related channelopathies: clinical manifestations and treatment options. Handb Exp Pharmacol. 2023;279:227–248. 10.1007/164_2022_625 10.1007/164_2022_625 36592223

[3] den Dunnen DV, Bemelmans V, van de Vlies, van de Warrenburg. Mutations in potassium channel KCND3 cause spinocerebellar ataxia type 19. Ann Neurol. 2012;72(6):870–80. 10.1002/ana.23700 23280838

[4] Parolin Schnekenberg R, Perkins EM, Miller JW, Davies WIL, D’Adamo MC, Pessia M, et al. De novo point mutations in patients diagnosed with ataxic cerebral palsy. Brain. 2015;138(7):1817–32. 25981959 10.1093/brain/awv117 PMC4572487

[5] Cunha P, Petit E, Coutelier M, Coarelli G, Mariotti C, Faber J, et al. Extreme phenotypic heterogeneity in non-expansion spinocerebellar ataxias. Am J Hum Genet. 2023;110(7):1098–109. 37301203 10.1016/j.ajhg.2023.05.009 PMC10357418

[6] Pellerin D, Danzi MC, Wilke C, Renaud M, Fazal S, Dicaire M-J, et al. Deep Intronic FGF14 GAA Repeat Expansion in Late-Onset Cerebellar Ataxia. N Engl J Med. 2023;388(2):128–41. 36516086 10.1056/NEJMoa2207406 PMC10042577

[7] Du X, Wang J, Zhu H, Rinaldo L, Lamar K-M, Palmenberg AC, et al. Second cistron in CACNA1A gene encodes a transcription factor mediating cerebellar development and SCA6. Cell. 2013;154(1):118–33. 23827678 10.1016/j.cell.2013.05.059 PMC3939801

[8] Ophoff RA, Terwindt GM, Vergouwe MN, Van Eijk R, Oefner PJ, Hoffman SMG, et al. Familial hemiplegic migraine and episodic ataxia type-2 are caused by mutations in the Ca2+ channel gene CACNL1A4. Cell. 1996;87(3):543–52. 8898206 10.1016/s0092-8674(00)81373-2

[9] Ali SR, Singh AK, Laezza F. Identification of amino acid residues in fibroblast growth factor 14 (FGF14) required for structure-function interactions with voltage-gated sodium channel Nav1.6. J Biol Chem. 2016;291(21):11268–84. 10.1074/jbc.M115.703868 PMC4900273 26994141

[10] Pablo JL, Pitta GS. FGF14 is a regulator of KCNQ2/3 channels. Proc Natl Acad Sci USA. 2017;114(1):154–9. 27994149 10.1073/pnas.1610158114 PMC5224356

[11] Pellerin D, Danzi MC, Renaud M, Houlden H, Synofzik M, Zuchner S, et al. Spinocerebellar ataxia 27B: A novel, frequent and potentially treatable ataxia. Clin Transl Med. 2024;14(1):e1504. 10.1002/ctm2.1504 10.1002/ctm2.1504 PMC10819088 38279833

[12] Indelicato E, Fleszar Z, Pellerin D, Nachbauer W, Zuchner S, Traschütz A, et al. GAA-FGF14 expansions and CACNA1A variants: phenotypic overlap and diagnostic implications. Mov Disord. 2025;40(10):2262–2268. 10.1002/mds.30328 10.1002/mds.30328 PMC12553994 40879304

[13] Indelicato E, Nachbauer W, Karner E, Eigentler A, Wagner M, Unterberger I, et al. The neuropsychiatric phenotype in CACNA1A mutations: a retrospective single center study and review of the literature. Eur J Neurol. 2019;26(1):66-e7. 30063100 10.1111/ene.13765

[14] Ashton C, Indelicato E, Pellerin D, Clément G, Danzi MC, Dicaire MJ, et al. Spinocerebellar ataxia 27B: episodic symptoms and acetazolamide response in 34 patients. Brain Commun. 2023;5(5):fcad239. 10.1093/braincomms/fcad239 10.1093/braincomms/fcad239 PMC10495284 37705681

[15] Chen S, Ashton C, Sakalla R, Clement G, Planel S, Bonnet C, et al. Involvement of the Superior Cerebellar Peduncles in GAA- FGF14 Ataxia. Neurol Genet. 2025;11(2):e200253. 10.1212/NXG.0000000000200253 10.1212/NXG.0000000000200253 PMC11849524 39996128

[16] Faber J, Kügler D, Bahrami E, Heinz LS, Timmann D, Ernst TM, et al. CerebNet: A fast and reliable deep-learning pipeline for detailed cerebellum sub-segmentation. Neuroimage. 2022;1:264. 10.1016/j.neuroimage.2022.119703 PMC9771831 36349595

[17] FerreiraM, Schaprian T, Kügler D, Reuter M, Deike-Hoffmann K, Timmann D, et al. Cerebellar Volumetry in Ataxias: Relation to Ataxia Severity and Duration. Cerebellum. 2024;23(4 ):1521–1529. 10.1007/s12311-024-01659-0 10.1007/s12311-024-01659-0 PMC11269395 38363498

[18] Reetz K, Costa AS, Mirzazade S, Lehmann A, Juzek A, Rakowicz M, et al. Genotype-specific patterns of atrophy progression are more sensitive than clinical decline in SCA1, SCA3 and SCA6. Brain. 2013;136(Pt 3):905–17. 23423669 10.1093/brain/aws369

[19] Faber J, Schaprian T, Berkan K, Reetz K, França MC, de Rezende TJR, et al. Regional brain and spinal cord volume loss in spinocerebellar ataxia type 3. Mov Disord. 2021;36(10):2273–81. 33951232 10.1002/mds.28610 PMC9521507

[20] Coarelli G, Dubec-Fleury C, Petit E, Sayah S, Fischer C, Nassisi M, et al. Longitudinal changes of clinical, imaging, and fluid biomarkers in preataxic and early ataxic spinocerebellar ataxia type 2 and 7 carriers. Neurology. 2024;103(5):e209749. 39133883 10.1212/WNL.0000000000209749 PMC11361831

[21] Dichgans M, Herzog J, Freilinger T, Wilke M, Auer DP. 1H-MRS alterations in the cerebellum of patients with familial hemiplegic migraine type 1. Neurology. 2005;64(4):608–13. 15728280 10.1212/01.WNL.0000151855.98318.50

[22] Zielman R, Teeuwisse WM, Bakels F, Van Der Grond J, Webb A, Van Buchem MA, et al. Biochemical changes in the brain of hemiplegic migraine patients measured with 7 tesla 1H-MRS. Cephalalgia. 2014;34(12):959–67. 24651393 10.1177/0333102414527016

[23] Richards S, Aziz N, Bale S, Bick D, Das S, Gastier-Foster J, et al. Standards and guidelines for the interpretation of sequence variants: A joint consensus recommendation of the American college of medical genetics and genomics and the association for molecular pathology. Genet Med. 2015;17(5):405–24. 25741868 10.1038/gim.2015.30 PMC4544753

[24] Bonnet C, Pellerin D, Roth V, Clément G, Wandzel M, Lambert L, et al. Optimized testing strategy for the diagnosis of GAA-FGF14 ataxia/spinocerebellar ataxia 27B. Sci Rep. 2023;13(1):9737. 10.1038/s41598-023-36654-8 10.1038/s41598-023-36654-8 PMC10272173 37322040

[25] Schmitz-Hübsch T, Du Montcel ST, Baliko L, Berciano J, Boesch S, Depondt C, et al. Scale for the assessment and rating of ataxia: Development of a new clinical scale. Neurology. 2006;66(11):1717–20. 16769946 10.1212/01.wnl.0000219042.60538.92

[26] Henschel L, Conjeti S, Estrada S, Diers K, Fischl B, Reuter M. FastSurfer - A fast and accurate deep learning based neuroimaging pipeline. Neuroimage. 2020;1(219):117012. 10.1016/j.neuroimage.2020.117012 PMC7898243 32526386

[27] ChenS, Ashton C, Sakalla R, Clement G, Planel S, Bonnet C, et al. Neuroradiological findings in GAA-FGF14 ataxia (SCA27B): more than cerebellar atrophy. medRxiv. 2024:2024.02.16.24302945. 10.1101/2024.02.16.24302945

[28] Rezende TJR, de Paiva JLR, Martinez ARM, Lopes-Cendes I, Pedroso JL, Barsottini OGP, et al. Structural signature of SCA3: From presymptomatic to late disease stages. Ann Neurol. 2018;84(3):401–8. 30014526 10.1002/ana.25297

[29] Eichler L, Bellenberg B, Hahn HK, Köster O, Schöls L, Lukas C. Quantitative assessment of brain stem and cerebellar atrophy in spinocerebellar ataxia types 3 and 6: impact on clinical status. AJNR Am J Neuroradiol. 2011;32(5):890–7. 21372168 10.3174/ajnr.A2387 PMC7965570

[30] Stefanescu MR, Dohnalek M, Maderwald S, Thürling M, Minnerop M, Beck A, et al. Structural and functional MRI abnormalities of cerebellar cortex and nuclei in SCA3, SCA6 and Friedreich’s ataxia. Brain. 2015;138(5):1182–97. 25818870 10.1093/brain/awv064 PMC5963415

[31] Fox PM, Malepati S, Manaster L, Rossignol E, Noebels JL. Developing a pathway to clinical trials for CACNA1A-related epilepsies: A patient organization perspective. Ther Adv rare Dis. 2024;1(5):26330040241245724. 10.1177/26330040241245725 PMC11047245 38681799

[32] Tottene A, Fellin T, Pagnutti S, Luvisetto S, Striessnig J, Fletcher C, et al. Familial hemiplegic migraine mutations increase Ca2+ influx through single human CaV2.1 channels and decrease maximal CaV2.1 current density in neurons. Proc Natl Acad Sci USA. 2002;99(20):13284. 10.1073/pnas.192242399 PMC130625 12235360

[33] Walter JT, Alviña K, Womack MD, Chevez C, Khodakhah K. Decreases in the precision of Purkinje cell pacemaking cause cerebellar dysfunction and ataxia. Nat Neurosci. 2006;9(3):389–97. 16474392 10.1038/nn1648

[34] RansdellJL, Brown SP, Xiao M, Ornitz DM, Nerbonne JM. In Vivo expression of an SCA27A-linked FGF14 mutation results in haploinsufficiency and impaired firing of cerebellar purkinje neurons. bioRxiv. 2024:2024.10.25.620253. 10.1101/2024.10.25.620253 10.1523/JNEUROSCI.2016-24.2026 PMC12925659 41558966

[35] Indelicato E, Boesch S. From genotype to phenotype: expanding the clinical spectrum of CACNA1A variants in the era of next generation sequencing. Frontier Neurol. 2021;12:639994 10.3389/fneur.2021.639994 PMC7960780 33737904

[36] Allen AS, Berkovic SF, Cossette P, Delanty N, Dlugos D, Eichler EE, et al. De novo mutations in epileptic encephalopathies. Nature. 2013;501(7466):217–21. 23934111 10.1038/nature12439 PMC3773011

[37] Pietrobon D. CaV2.1 channelopathies. Pflugers Arch. 2010;460(2):374–93. 10.1007/s00424-010-0802-8 20204399

[38] Chang SY, Yong TF, Yu CY, Liang MC, Pletnikova O, Troncoso J, et al. Age and gender-dependent alternative splicing of P/Q-type calcium channel EF-hand. Neuroscience. 2007;145(3):1026–36. 17291689 10.1016/j.neuroscience.2006.12.054 PMC1978091

