# Supplementary material for: Genotype and Age at Onset Drive Vermis Atrophy in CACNA1A- and GAA-FGF14-related Ataxias
Source: Cerebellum. 2026 Mar 4;25(2):29. doi: 10.1007/s12311-026-01966-8 (PMC12956909; doi:10.1007/s12311-026-01966-8)
Supplement: Supplementary file 1 — Supplementary file1 (DOCX 19 KB) [file 12311_2026_1966_MOESM1_ESM.docx]

**Supplementary table**

Detailed molecular and clinical characteristics of the study cohort.

The column “Subjects” reports the numeric identifier from figure 1. The column “Genotype” reports the detected *CACNA1A* variant along with the corresponding transcript or the GAA•TTC repeat lengths in patients with GAA-*FGF14*-related ataxia. SCA27B: Spinocerebellar ataxia 27B.
